# A Rare Presentation of Hyperphagia and Parasomnias Associated With Chromosome 4q Deletion: A Case Report

**DOI:** 10.1155/crps/5061704

**Published:** 2025-04-01

**Authors:** Sarah L. Vaithilingam, Sheldon R. Garrison, Aman Mahajan, Julia F. Kranz, John T. Diener

**Affiliations:** ^1^Child and Adolescent Inpatient Unit, Rogers Behavioral Health, Brown Deer, Wisconsin, USA; ^2^Research Center, Rogers Behavioral Health, Oconomowoc, Wisconsin, USA; ^3^Department of Pediatrics, Medical College of Wisconsin, Milwaukee, Wisconsin, USA

**Keywords:** attention-deficit/hyperactivity disorder (ADHD), autism, autism spectrum disorder (ASD), hallucinations, hyperphagia, parasomnia, rare disease, sleep

## Abstract

**Background:** Chromosome 4q deletion is a rare genetic disorder affecting an estimated 1 out of 100,000 people. It is characterized by microdeletions of the long arm of chromosome 4 with variable clinical presentations including heart defects, craniofacial and skeletal abnormalities, short stature, and developmental delays. While behavioral and psychiatric symptoms have been reported in a small number of patients with chromosome 4q deletions, none of these reports have described the hyperphagia or parasomnia symptoms that are presented in the current case.

**Case Presentation:** A 7-year-old boy presented with a microdeletion of the long arm of chromosome 4 that resulted in psychiatric symptoms and neurodevelopmental delays. Notable manifestations included hyperphagia and parasomnias, in addition to aggression, functional encopresis, and speech delays. The boy's initial treatment was markedly delayed due to limited genetic testing at the age of 1 year, which led to a misdiagnosis of childhood aggression. This limited the care team involvement for neurologic evaluation and appropriate school interventions that would have otherwise been indicated. At inpatient admission, a multidisciplinary approach to diagnosis and treatment was adopted, encompassing pharmacological and behavioral interventions. The patient's attention-deficit/hyperactivity disorder (ADHD) was treated, and his individualized education plan included a functional behavioral assessment, as well as occupational therapy and speech and language services. Following a 4-day inpatient stay, the patient demonstrated a significant decrease in aggressive behaviors.

**Conclusion:** Chromosome 4q deletion-related behaviors parallel those of children with autism spectrum disorder (ASD), and treatment is primarily focused on behavioral interventions. To successfully manage the psychiatric features of this complex condition, the involvement of a multidisciplinary team is recommended.

## 1. Introduction

Chromosome 4q deletion is a rare genetic finding marked by terminal or interstitial deletions on the long arm of chromosome 4. It is estimated to occur in 1 out of 100,000 people and affects males and females equally [1]. Chromosome 4q deletion is characterized by a vast range of phenotypic presentations, including developmental delays, short stature, congenital heart defects, and facial abnormalities. It also has varied psychiatric presentations including developmental delay, attention-deficit/hyperactivity disorder (ADHD), autism spectrum disorder (ASD), and schizoaffective disorder. The large phenotypic variation of chromosome 4q deletion makes it challenging to characterize specific genotype–phenotype relationships for varying 4q deletions in children with psychiatric symptoms [2]. The rarity of chromosome 4q deletions and the incomplete penetrance of its symptoms have been highlighted as major obstacles in outlining candidate genes for specific phenotypes [2, 3].

In the present report, a child with 4q deletion presented with aggressive behavior, parasomnias, cognitive impairment, speech disorder, functional encopresis, and hyperphagia. While aggression, intellectual disability, and speech delay have been previously described in the literature on 4q microdeletions, parasomnias and hyperphagia have not. The maladaptive behaviors in this case were linked to sleep-related hallucinations, hyperphagia, and food-seeking behaviors.

## 2. Case Presentation

A 7-year-old boy presented to the child and adolescent inpatient psychiatric unit in a large behavioral health care system with aggressive behaviors, hyperphagia, and parasomnias. He was born to nonconsanguineous parents, and there were no complications during pregnancy or delivery. Prior to hospitalization, the patient did not undergo any psychiatric evaluations, and there was no history of home medications.

The patient had a 7-year medical history of developmental delays and behaviors, including hyperphagia, encopresis, speech, and articulation problems and problems with fine motor skills. His history included concerns of malnutrition and suspected failure to thrive at the age of 1 year. At that time, a nasogastric tube was initiated due to concerns of failure to thrive. Routine lab work did not show any abnormalities. Following weeks without gaining weight, and due to the complexity of the presentation, genetic testing was performed to determine genetic disease involvement. This initial testing revealed a microdeletion of chromosome 4, and the patient was subsequently diagnosed with a chromosome 4q microdeletion. Further genetic analysis determined that his mother was the carrier.

At the time of admission, the 7-year-old patient's general physical examination showed short stature, 25th percentile for weight, microcephaly, gastroesophageal reflux disease, lactose intolerance, and unspecified neurocognitive disorder. Other psychiatric complaints included parasomnia, particularly hypnogogic hallucinations, where he often described the “snakes and monsters” that would attempt to eat him. The patient was raised by a single parent who reported that these daily hallucinations would cause the patient to lock himself in the bathroom screaming and crying. Additionally, he had hyperphagia that led to atypical behaviors. His food-seeking behaviors were excessive, with his parent reporting that he would hoard food, consume large quantities of food to the point of emesis, consumed food throughout the night and day, and was reported to obtain discarded food items taken from the outdoors.

A connection between the hyperphagia and aggressive behaviors was suspected, particularly at times when his demands for food were not met. These behaviors were at times dangerous to the patient and those around him. For example, his parent reported that he intentionally set the microwave on fire when his food demands were not met. The patient also had daily parasomnias, as defined by the International Classification for Sleep Disorders [4]. This would cause significant behavioral dysregulation. The patient's parent reported that at home he would also urinate in cups and hide them in his room.

The patient's parent also provided historical reports of aggressive behaviors that included flipping tables, pulling his sibling out of bed, and burning the targeted sibling. At the time of admission for psychiatric hospitalization, the patient demonstrated physically aggressive behaviors toward objects and others, selective mutism, and poor eye contact. The patient also regularly displayed self-injurious behaviors that included punching himself or repeatedly hitting his head against the wall to the point he would bleed. The patient continued to urinate in inappropriate places in the hospital, typically in the corner of his room and in cups.

After psychiatric inpatient admission, the patient was evaluated and stabilized for acute aggression. Given the neurodevelopmental and behavioral concerns, much of the treatment course focused on presenting symptoms and setting up an appropriate medical workup including cardiology, child development, gastroenterology (for hyperphagia and encopresis), sleep medicine for non-rapid eye movement (REM) sleep disorder, and neurocognitive evaluation. Community support that included a case manager, therapist, and psychiatrist was also established in advance of discharge. Poor reality testing was not initially demonstrated, and therefore, he was not diagnosed with psychosis or started on antipsychotic medications. The patient was diagnosed with other specified neurodevelopmental disorder, ADHD, and non-REM sleep disorder. Due to concerns that a stimulant medication may cause irritability, he was instead prescribed clonidine extended-release 0.1 mg twice daily (b.i.d.), to manage his sleep, impulsivity, and aggression. The patient received daily behavioral group therapy, which was tailored to children and included positive reinforcement.

The patient was discharged following 4 days of treatment after demonstrating decreased aggressive and self-injurious behaviors. He denied experiencing perceptual disturbances, including parasomnias, and was calm and cooperative. He continued to present with ASD-related symptoms. He had poor eye contact and was easily distracted by objects in the room. His verbal skills were limited, using three- to five-word sentences. He described his mood as “good” although his affect appeared blunted, with minimal facial expression. Thought content reflected questions about animals and when he would be able to talk to his mother. His thought process was concrete and underdeveloped for chronological age. He had very little insight into why he was at the hospital, and his judgment was limited.

After discharging, the patient continued to be followed by child psychiatry and pediatric specialists. Further neurodevelopmental evaluation confirmed the diagnoses ADHD and other specified neurodevelopmental disorder. Cardiology examinations did not find any concerns for structural or electrical abnormalities that could affect the prescribing of atypical antipsychotics for treating his aggressive behaviors. Recommendations to address the hyperphagia, parasomnias, and other symptoms following discharge included continuation on 0.1 mg clonidine b.i.d., intelligence quotient (IQ) testing, functional behavioral assessment, speech and language assessment, occupational therapy, consult with a pediatric dietitian or nutritionist, and completion of a sleep study.

## 3. Discussion

This case report is focused on a unique presentation of chromosome 4q deletion. To the best of our knowledge, there are no previous reports of the association of parasomnia and hyperphagia with chromosome 4q deletion. The patient's preoccupation with food and problems with REM sleep are suspected to have contributed to the complexity of his behavioral presentation, particularly his aggression, which became difficult to quell without psychiatric intervention. Importantly, adverse food-related behaviors are not uncommon in children; however, the extent to which this child was behaving around food was comparable to children with Prader–Willi syndrome [5]. Collectively, these features placed substantial burden on both the patient and his family.

Adding to this complexity, the correlation between cognitive impairment and the psychiatric symptoms observed in the reported case remains unclear. Moreover, the severity of the intellectual disability varies broadly in individuals with confirmed 4q deletions. Consequently, a critical avenue for future investigation entails examining the interplay between intellectual disabilities and the diverse array of psychiatric features within this patient population.

The large phenotypic variation of chromosome 4q deletion makes it challenging to characterize specific genotype–phenotype relationships for varying 4q deletions in children with psychiatric symptoms such as developmental delay, ASD, ADHD, and schizoaffective disorder. Developmental delay, which included language, encopresis, and fine motor skills in our patient, is a particular phenotype of chromosome 4q deletion that has been closely evaluated in critical regions in the chromosome to determine the genotype–phenotype relationship [6]. Deletion of the terminal region of the long arm of chromosome 4 results in common features of chromosome 4q deletion, including developmental delay, learning difficulties, and craniofacial, digital, skeletal, and cardiac anomalies [7]. Phenotypic differences between patients with a terminal deletion seems to largely be based on breakpoint locations starting at 4q33 to 4q34, which is suggested to be the critical region for the 4q terminal deletion [6, 7].

The genetic underpinnings of ASD presentation in children with chromosome 4q deletion continue to be explored, with some genotype–phenotype correlations previously reported. For example, a 19 MB deletion spanning from 4q32 to 4q34 was identified in a young boy with chromosome 4q deletion and resulting ASD [8]. By examining the deletion region and associated genes, five candidate autism-specific genes were identified [8]. One goal of this line of work is to continue to individualize treatment planning. The patient in the current case report had limited verbal expression, which limited the assessment and identification of his social impairment. To address this limitation, neuropsychological testing for autism was recommended to enhance the depth of understanding of the patient's social reciprocity, despite his language deficits. Specifically, it was recommended that either the Autism Diagnostic Observation Schedule (ADOS) [9] or the Autism Diagnostic Interview-Revised (ADI-R) [10] be used.

ADHD is also common in children with chromosome 4q microdeletions. In a study of a multigenerational family who was genetically isolated, linkage was determined at certain chromosomal regions, including 4q13.2. Results were consistent across multiple forms of linkage analysis and indicate that chromosome 4 does contain ADHD susceptibility genes [11]. ADHD is often associated with the dopamine transporter 1 gene (DAT1) that has been mapped to chromosome 5p15. It codes for a solute carrier protein responsible for the reuptake of dopamine from the synaptic cleft back to the presynaptic neuron. Other genes such as mastermind like transcriptional coactivator 3 (MAML3; chromosomal 4 at 4q28.3) and the dopamine receptor D5 (DRD5; chromosome 4 at 4p15.3) have been explored as potential contributors to ADHD and disruptive behavioral disturbances [12, 13]. However, their role in the presentation of chromosome 4q deletion is not well understood.

Studies have also begun to explore the potential presentation of schizoaffective disorder in patients with chromosome 4q deletion. This relationship was first described in an individual with a 4q deletion who had a learning disability and psychosis [14]. It has been established that those with intellectual disabilities may be at a significantly higher risk of developing schizoaffective disorder [15]. Indeed, to help improve the lives of patients diagnosed with cognitive impairment, screening for symptoms of schizophrenia, bipolar disorder, and other mental health conditions may be best practice [14, 16]. It remains uncertain whether individuals with 4q deletions and associated intellectual disabilities are at increased risk for these conditions.

The challenging range of psychiatric symptoms faced by patients with chromosome 4q deletion, and their families and caregivers, can be substantial. Parents and guardians of children diagnosed with 4q deletion have reported feeling overwhelmed and concerned about the dearth of information and training on the condition that is available to medical professionals [1]. Delayed genetic testing is common for rare diseases, which can subsequently delay comprehensive psychiatric testing and optimal therapeutic approaches. It is important for additional studies to be published that continue to characterize this debilitating condition. With an estimated 3300 people diagnosed in the United States alone [1], yet fewer than 200 cases reported worldwide [2], there is likely to be a significant number of undiagnosed patients, highlighting an urgent need to inform the broader medical community.

**Figure 1:**
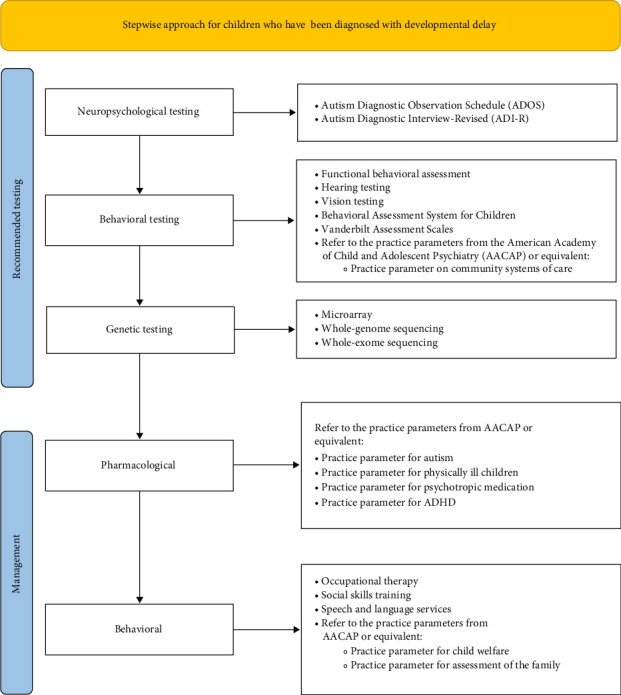
Flowchart of the recommended testing and management for children presenting with developmental delays when genetic disease is suspected that may increase the detection of chromosome 4q deletion.

Without a clear constellation of psychiatric symptoms that define 4q deletion, identifying patients with this condition remains challenging. This specific case contributes to the characterization of chromosome 4q deletion, as the patient had a unique psychiatric presentation that included hyperphagia and parasomnia. Once the diagnosis is made, and aside from crisis stabilization in an acute psychiatric setting, we strongly recommend an individualized education plan that includes a functional behavioral assessment, the Behavioral Assessment System for Children [17], the National Institute for Children's Health Quality (NICHQ) Vanderbilt Assessment Scales [18], occupational therapy, social skills training, and speech and language services (Figure 1). To successfully manage the psychiatric features of this complex condition, we also recommend the involvement of a multidisciplinary team that includes a psychiatrist, pediatrician, medical geneticist, dietician, speech and language therapist, physical therapist, occupational therapist, psychologist, and special education teacher to reduce the need for crisis intervention.

## Data Availability

Data are available on request due to privacy/ethical restrictions.

## References

[1] Strehle E. M., Middlemiss P. M. (2007). Children with 4q-Syndrome: The Parents’ Perspective. *Genetic Counseling (Geneva, Switzerland)*.

[2] Strehle E. M., Yu L., Rosenfeld J. A. (2012). Genotype-Phenotype Analysis of 4q Deletion Syndrome: Proposal of a Critical Region. *American Journal of Medical Genetics Part A*.

[3] Vona B., Nanda I., Neuner C. (2014). Terminal Chromosome 4q Deletion Syndrome in An Infant With Hearing Impairment and Moderate Syndromic Features: Review of Literature. *BMC Medical Genetics*.

[4] Thorpy M. (1990). *Diagnostic Classification Steering Committee: International Classification of Sleep Disorders: Diagnostic and Coding Manual*.

[5] Butler M. G., Miller J. L., Forster J. L. (2019). Prader-Willi Syndrome—Clinical Genetics, Diagnosis and Treatment Approaches: An Update. *Current Pediatric Reviews*.

[6] Vlaikou A. M., Manolakos E., Noutsopoulos D. (2014). An Interstitial 4q31.21q31.22 Microdeletion Associated with Developmental Delay. *Cytogenetic and Genome Research*.

[7] Kaalund S. S., Moller R. S., Teszas A. (2008). Investigation of 4q-Deletion in Two Unrelated Patients Using Array CGH. *American Journal of Medical Genetics Part A*.

[8] Ramanathan S., Woodroffe A., Flodman P. L. (2004). A Case of Autism with An Interstitial Deletion on 4q Leading to Hemizygosity for Genes Encoding for Glutamine and Glycine Neurotransmitter Receptor Sub-Units (AMPA 2, GLRA3, GLRB) and Neuropeptide Receptors NPY1R, NPY5R. *BMC Medical Genetics*.

[9] Lord C., Rutter M., Goode S. (1989). Autism Diagnostic Observation Schedule: A Standardized Observation of Communicative and Social Behavior. *Journal of Autism and Developmental Disorders*.

[10] Lord C., Rutter M., Le Couteur A. (1994). Autism Diagnostic Interview-Revised: A Revised Version of a Diagnostic Interview for Caregivers of Individuals with Possible Pervasive Developmental Disorders. *Journal of Autism and Developmental Disorders*.

[11] Arcos-Burgos M., Castellanos F. X., Pineda D. (2004). Attention-Deficit/Hyperactivity Disorder in a Population Isolate: Linkage to Loci at 4q130.2, 5q330.3, 11q22, and 17p11. *The American Journal of Human Genetics*.

[12] Demontis D., Walters R., Rajagopal V. M. (2019). Identification of Risk Variants and Characterization of the Polygenic Architecture of Disruptive Behavior Disorders in the Context of ADHD. *bioRxiv*.

[13] Wu J., Xiao H., Sun H., Zou L., Zhu L. Q. (2012). Role of Dopamine Receptors in ADHD: A Systematic Meta-Analysis. *Molecular Neurobiology*.

[14] Pickard B. S., Hollox E. J., Malloy M. P., Porteous D. J., Blackwood D. H., Armour J. A. (2004). Muir WJ: A 4q35.2 Subtelomeric Deletion Identified in a Screen of Patients with Co-Morbid Psychiatric Illness and Mental Retardation. *BMC Medical Genetics*.

[15] Lakhan R. (2013). The Coexistence of Psychiatric Disorders and Intellectual Disability in Children Aged 3-18 Years in the Barwani District, India. *ISRN Psychiatry*.

[16] Carmeli E., Imam B. (2014). Health Promotion and Disease Prevention Strategies in Older Adults with Intellectual and Developmental Disabilities. *Frontiers in Public Health*.

[17] Reynolds C. R., Kamphaus R. W., Vannest K. J. (2015). *BASC3: Behavior Assessment System for Children*.

[18] Wolraich M. L., Lambert W., Doffing M. A., Bickman L., Simmons T., Worley K. (2003). Psychometric Properties of the Vanderbilt ADHD Diagnostic Parent Rating Scale in a Referred Population. *Journal of Pediatric Psychology*.

[19] Vaithilingam S., Garrison S., Mahajan A., Krantz J., Diener J. (2023). *A Novel Psychiatric Phenotype of Chromosome 4q Deletion: A Case Report*.

